# Case Report: Metastatic succinate dehydrogenase–deficient gastrointestinal stromal tumor treated with chemotherapy and immune checkpoint inhibitor

**DOI:** 10.3389/fonc.2025.1549232

**Published:** 2025-03-10

**Authors:** Arthur Claessens, Jérôme Edouard Plaza, Jean-Yves Blay

**Affiliations:** ^1^ Department of Medical Oncology, Institut de Cancérologie de Lorraine, Vandœuvre-lès-Nancy, France; ^2^ Department of Medical Oncology, Hôpital Robert Schuman - UNEOS Groupe Hospitalier Associatif, Vantoux, France; ^3^ Department of Medical Oncology, Centre Léon Bérard, Lyon, France; ^4^ Unicancer, Paris, France

**Keywords:** gastrointestinal stromal tumor (GIST), immunotherapy combined therapy, chemotherapy - oncology, young adult, long responders, complete response (CR)

## Abstract

We report a young adult with a recurrent metastatic succinate dehydrogenase (SDH)–deficient gastrointestinal stromal tumor (GIST) achieving a pathological complete response with a chemoimmunotherapy regimen. The patient underwent a gastroscopy, which revealed a large-ulcerated tumor on the greater gastric curvature. Histological analysis showed a poorly differentiated SDHB-deficient GIST with PDL1 expression at a 100% rate and a RET gene fusion. Rapid metastatic relapse after subtotal gastrectomy was resistant to imatinib. The patient received four cycles of cisplatin, etoposide, and pembrolizumab followed by pembrolizumab maintenance with a complete response. Late relapse 3 years later was treated with the same regimen and achieved complete pathological response. Two years later, an isolated focus was removed, showing the recurrent SDH-deficient GIST. Pembrolizumab is still in maintenance with no relapse to date. The role of chemoimmunotherapy as part of treatment in recurrent metastatic SDH-deficient, PDL1-positive GIST patients is worth further investigation.

## Introduction

Gastrointestinal stromal tumors (GISTs) are prevalent malignant mesenchymal tumors of the gastrointestinal tract, with most cases associated with mutations in the KIT or PDGFRA genes. Ten percent of patients present with SDH-deficient GIST, lacking identifiable mutations and having limited therapeutic options.

So far, immune checkpoint inhibitors (ICIs) have been used with limited success in soft-tissue sarcoma. Here, we present a case report of a young adult with an SDH-deficient GIST refractory to tyrosine kinase inhibitors who achieved a complete response to treatment combining chemotherapy and immunotherapy.

## Case presentation

A young adult with a background of atopic dermatitis and chronic gastritis associated with Helicobacter pylori presented in their early ‘20s with recurrent episodes of iron-deficiency anemia requiring blood transfusions. A gastroscopy revealed a large-ulcerated tumor of the greater gastric curvature, causing bleeding. A subsequent CT scan revealed a locally advanced lesion with interruption of the gastric wall. Histological analysis of biopsies identified a poorly differentiated tumor expressing CK7, with infiltration of the gastric wall. A semi-emergency subtotal gastrectomy was performed to control the bleeding, confirming infiltration of the gastric wall by a poorly differentiated tumor, with no tertiary lymphoid structures involved. The pathology review from a national reference center determined the GIST nature of the tumor based on CD34 and DOG1 expression, with large dedifferentiated areas that lacked these markers. PDL1 expression was assessed at 100%, and the mitotic index was high (143/5 mm^2^) in dedifferentiated areas. Next-generation sequencing (NGS) revealed no KIT or PDGFRA mutations but identified a succinate dehydrogenase (SDH) mutation in subunit B and an RET gene fusion.

**Figure 1:**
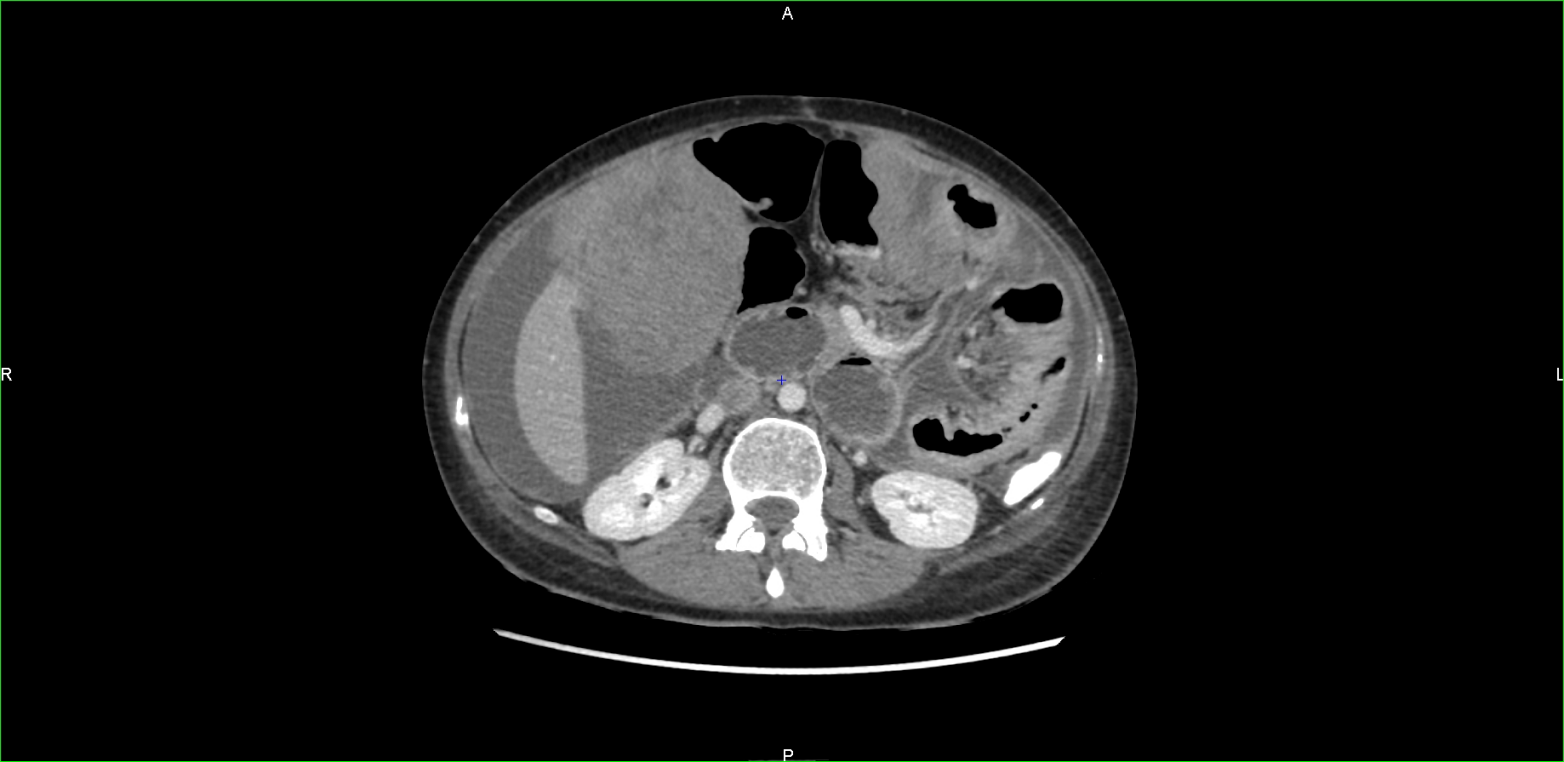
CT scan showing early and massive relapse.

Treatment with imatinib was initiated because of suspect lesions on an early CT scan ordered for abdominal discomfort. Three months later, as the patient suffered refractory intestinal obstruction, abdominal pain, and deterioration in general condition, imaging was obtained showing rapid progression with peritoneal and liver metastases associated with tumoral ascites (**Figure 1**).

**Figure 2:**
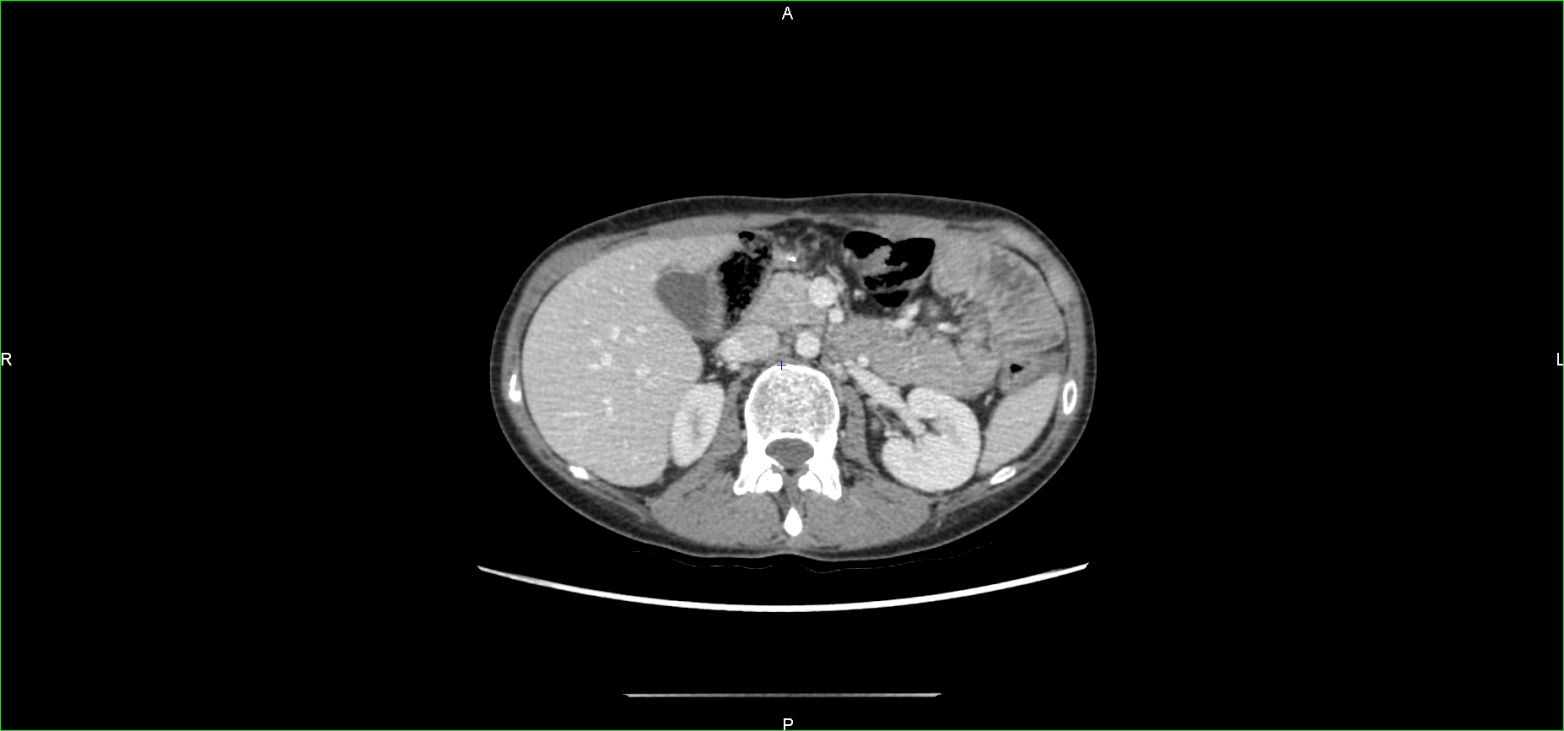
CT scan after combined treatment with chemotherapy and immunotherapy.

A multidisciplinary medical board stated that temozolomide as a palliative treatment should not be used in this setting and proposed a second-line therapy with four cycles of treatment combining chemotherapy and immunotherapy with cisplatin 100 mg/m^2^, etoposide 100 mg/m^2^ and pembrolizumab 200 mg every 3 weeks based on the predominantly dedifferentiated nature of the GIST and the massive and early nature of the relapse. A sepsis related to Escherichia coli and Klebsiella pneumoniae urinary tract infection developed after the first administration of chemoimmunotherapy, leading to admission in a critical care unit. The outcome was favorable after antibiotic treatment, allowing the treatment protocol to be continued. The treatment led to complete remission according to RECIST 1.1 for 3 years with continued immunotherapy without interruption (**Figure 2**).

Three years later, when a lesion appeared adjacent to the left colon, a follow-up CT scan carried out at 3 months showed that the lesion had progressed, measuring 46 mm × 35 mm, and that a second lesion measuring 47 mm × 42 mm had appeared. In view of the hypermetabolic nature of the PET scan and the rapid progression of these lesions, a medical board recommended that biopsy be omitted with preferring to resume chemotherapy with continued pembrolizumab. Rechallenge with three cycles of cisplatin 100 mg/m^2^, etoposide 100 mg/m^2^, and pembrolizumab 200 mg, followed by surgery, demonstrated complete response on anatomical pathology. In a context of a 2-year disease-free condition, and according to patient preference, maintenance of pembrolizumab 400 mg with a 6-week cycle started. Immunotherapy tolerance in this patient was suitable, with mild transient asthenia and nausea during the 2 days following administration.

**Figure 3:**
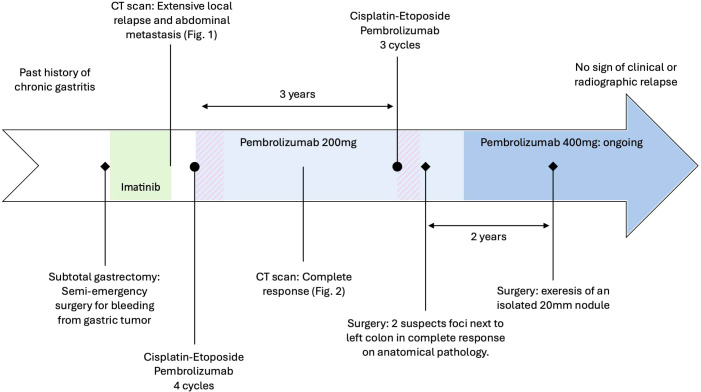
Patient timeline with relevant data from the episode of care.

Two years later, an isolated hypermetabolic 16 mm para-gastric focus was detected on PET scan. The nodule was removed, showing recurrence of an SDH-deficient GIST. A consultation at a national reference center proposes a continuing immunotherapy treatment of intravenous pembrolizumab 400 mg and close radiologic and clinical monitoring. The RET gene fusion may be activated in the event of relapse. To date, the patient has no functional limitations and is asymptomatic, with no radiographic relapse (**Figure 3**).

## Discussion

Treatment with imatinib has drastically altered the prognosis of patients with adjuvant and metastatic GIST. However, 10% of patients do not show KIT and PDGFRA gene mutations and are categorized as wild-type or SDH-deficient GIST. This histological subtype predominantly affects young female patients with gastric involvement and commonly presents with metastatic disease that progresses rapidly. SDH-deficient GISTs are generally indolent, and treatment options are limited in case of recurrence or incomplete surgical resection, as the response to tyrosine kinase inhibitors is usually compromised (1). This case report underlines the great clinical outcome in metastatic SDH-deficient GIST. The primary resistance to imatinib prompts us to take advantage of this histological setting to explore other possibilities. Although chemotherapy is generally poorly effective for the treatment of GIST, the rationale for using cisplatin and etoposide in this patient relies on the poorly differentiated nature of this SDH-deficient GIST. Rechallenge after 2 years with the same chemotherapies is also due to the initial poorly differentiated nature of the GIST and rapidly progressive recurrent disease (2).

Consideration of temozolomide in this context is linked to molecular characteristics of SDH-deficient GIST. Epigenetic inactivation of the enzyme O6-methylguanine-DNA methyltransferase (MGMT) via promoter methylation has been associated with the efficacy of alkylating agents in some tumors. SDH-deficient GISTs generally show significant DNA methylation, supporting the use of alkylating agents specifically targeting these small subgroups of GIST, with little clinical evidence and limited effect (3). This treatment, proposed as a palliative approach, was not retained in view of the patient’s young age and the limited expected therapeutic effect. The 2023 REGISTRI trial proposes to evaluate regorafenib in first-line metastatic disease in SDH-deficient GIST, with an amendment to include patients previously treated with imatinib. Treatment with regorafenib in 15 patients showed a favorable disease control rate, making it a potential first-line treatment option for recurrent or metastatic treatment for SDH-deficient GIST (4). An anti-RET drug such as selpercatinib is approved for the treatment of patients with RET mutations in thyroid cancer and non-small cell lung cancer with RET fusion. Compassionate access may be considered in the context of RET fusion without prejudging clinical outcomes in SDH-deficient GIST. These two approaches were not available at the time of metastatic relapse of this patient and could be considered as an option for a third-line treatment in case of relapse for this patient.

For this patient, initiation and maintenance of pembrolizumab was based on *in vitro* observation and the PDL1 expression rate of 100% in this SDH-deficient GIST. In view of this significant and prolonged response, it was decided to continue immunotherapy until inoperable relapse. The first attempts at immunotherapy in GIST date back to the early 2000s with the use of concurrent radiochemotherapy followed by the administration of OK432, a pharmaceutical preparation of Streptococcus pyogenes to activate neutrophils, natural killer cells, and macrophages (5). Since the advent of ICIs in the 2010s and the development of numerous tumor types, preclinical and clinical trials have focused on GIST and soft-tissue sarcoma. A strong *in-vitro* rationale supports the use of ICIs to treat GIST. A rich microenvironment in immune cells with activation of CD8+ T cells, inhibition of tumoral cell proliferation, and promotion of apoptosis suggests that ICIs could be a promising treatment option for these patients (6). Due to contradictory clinical results, it is difficult to rule on the usefulness of ICIs in GIST. Studies evaluating the safety and efficacy of ICIs included patients with multi-treated or metastatic GIST but did not specifically address the subgroup of SDH-deficient GIST due to the scarcity of this tumor subtype. Phase II studies have shown acceptable tolerability in these patients. Consequently, the use of ICIs can be considered, whereas it cannot be seen as a standard of care (7). According to the PEMBROSARC phase II trial, pembrolizumab associated with low-dose cyclophosphamide can be an option in soft-tissue sarcomas, including GIST, when intratumoral tertiary lymphoid structures are involved with the primary objective of a 6-month non-progression rate being met. It should be noted that no PDL1 expression threshold has been established for the potential use of ICIs in this setting (8). According to the ALLIANCE A091401 phase II study, no response was observed with nivolumab or nivolumab and ipilimumab. Enrolment was discontinued for futility due to lack of benefit. GISTs remain heterogeneous and generally cold tumors with limited immune infiltrates, but this study did not reveal whether SDH-deficient or wild-type GISTs were present in the sample (9). Another phase II study also reported non-significant responses in the nivolumab or ipilimumab and nivolumab arms in refractory GIST tumors. The wild-type GIST group does not appear to benefit preferentially from this therapeutic strategy (10). The association between wild-type GIST and response to immunotherapy was reported with an overall decrease in liver metastatic lesions during 64 cycles of treatment with nivolumab, followed by a progressive recovery after 71 cycles in a 40-year-old patient with good tolerance. An analysis of the tumor microenvironment showing a low CD3+ T cell infiltrate and PDL1 expression raises questions about the prognostic factors for response to immunotherapy in wild-type GIST (11). More recently, a case report of a woman with a metastatic SDH-deficient GIST, who had not responded to multiple previous treatments (imatinib, sunitinib, trametinib/ribociclib), was enrolled in the CA209-538 trial. The patient received an induction treatment of four doses of nivolumab (3 mg/kg) and ipilimumab (1 mg/kg), followed by 2 years of maintenance treatment with nivolumab. Over time, the patient achieved a progressively notable partial response according to RECIST 1.1 criteria (12).

Many approaches are currently being explored to treat patients with GIST, with a particular interest in targeting molecular alterations and pathways and combined approaches with tyrosine kinase inhibitors, small molecules, and immunotherapies (13). To our knowledge, this case illustrates the first reported case of successful chemoimmunotherapy in recurrent metastatic SDH-deficient GIST, leading to prolonged response. The use of cisplatin, etoposide, and pembrolizumab in this setting remains experimental and ideally should be considered only in the context of a clinical trial. Therefore, the role of chemoimmunotherapy as part of treatment in recurrent metastatic SDH-deficient, PDL1-positive GIST patient is worth further investigation.

## Data Availability

The original contributions presented in the study are included in the article/supplementary material. Further inquiries can be directed to the corresponding author.
